# In memory of the founding editor Prof. Dr. med. Ralf Herbert Gahr, MHBA

**DOI:** 10.3205/iprs000145

**Published:** 2020-09-08

**Authors:** Mohamed Ghanem, Christoph-Eckhard Heyde

**Affiliations:** 1 Department of Orthopedics, Traumatology and Plastic Surgery, University Hospital Leipzig, Germany

## Orbituary

It was with great dismay that we received the news of the death of our colleague Prof. Dr. Ralf Herbert Gahr, who passed away on August 2^nd^, 2020. Professor Gahr has served medicine as a passionate trauma surgeon for decades and shaped many colleagues as a visionary.

Prof. Dr. Ralf Gahr, born on June 14^th^, 1952 in Dortmund, studied human medicine in Cologne and London from 1970 to 1976. He obtained the medical doctorate degree in 1976. After completing his training as a specialist in surgery at the Dortmund Municipal Clinic, he specialized in the field of trauma surgery and was appointed senior physician at the Dortmund Trauma Clinic in 1986. His broad professional interest was reflected in his specialist qualification for surgery and the additional qualification in traumatology in the acquisition of the qualification for special trauma surgery, special hand surgery and surgical intensive medicine.

For decades, Prof. Gahr was very committed to the rescue service, first as an emergency doctor, later for many years as the chief emergency doctor. In 1993 he was awarded the Silver Badge of Honor by the Medical Board of LÄK-Westfalen-Lippe for the establishment of the emergency doctor training in North Rhine-Westphalia and for participating in the conception and development of the structures of chief emergency doctors (LNA) in Germany.

In 1993, Prof. Gahr took over the management of the Clinic for Trauma and Reconstructive Surgery at the St. Georg City Hospital in Leipzig. Here, we got to know him as a colleague who was sparkling for action and visions, who expanded and modernized the clinic with energy, diligence and perseverance and was one of the first in Germany to set up an interdisciplinary trauma center. Despite his wealth of professional and operational experience at that time, he was always keen on further developments in his field. In addition to regular advanced training, symposia and courses that he organized, his regard to interdisciplinarity and the goal of comprehensive patient treatment from the pre-clinical phase to optimal clinical care and follow-up care should be emphasized. Professor Gahr headed the interdisciplinary trauma center at the St. Georg City Hospital in Leipzig until his retirement in 2016. Many colleagues were trained at this renowned clinic under his leadership.

Figure 1 (Fig. 1)

Professor Gahr was scientifically active throughout his professional life. In 1997 he completed his habilitation at the University of Leipzig. In 2000 he was awarded an honorary professorship at the University of Skopje in Macedonia and in 2008 he was appointed Professor at the University of Leipzig. 

He enriched medical science as the editor of various specialist books, with numerous book contributions, a long series of publications and over 500 lectures at national and international scientific events. In addition, he was co-founder of the PubMed-listed journal “GMS Interdisciplinary Plastic & Reconstructive Surgery DGPW” in 2012 and has been editor-in-chief since then. Professor Gahr was involved in various editorial boards and as a reviewer for renowned specialist journals.

His professional commitment was great. He was a member and in the committees of various national and international professional societies in various responsible offices. For example, he has been a board member of the DGPW (German Society for Plastic and Reconstructive Surgery) since 2008, president of this society in 2010 and its general secretary since 2011. 

Professor Gahr has committed himself for the interests of the employer’s liability insurance association throughout his professional life. As a consulting doctor and expert, he was a sought-after expert here. His extensive experience in expert opinion led logically to the establishment of a sought-after medical expert institute.

His medical achievements and accomplishments are countless, examples include his medical commitment at the University of Skopje and in Lebanon, numerous awards and prizes, presidencies and board activities as well as his participation in international expert committees. The successful completion of a Master in Health Business Administration in 2014, among other things, testifies to his tireless ongoing interest, also across disciplines.

However, it is particularly important to us to pay tribute to the person Ralf Gahr. His humanistic education, his interest in art, literature and music, his long and successful career as a judoka, but also his fun at driving beautiful and fast cars are inseparable from this humorous, interesting and interested interlocutor. Moreover, for everyone who was trained by him or who worked with him, he remained a reliable and always present contact person and advisor for a lifetime.

We would like to express our condolences to his wife, who has stood by him for a lifetime, and to his children, both of whom have successfully embarked on a medical career, and wish them all the best and lots of strength. Even if it is difficult in the grief, they can proudly look back on the fulfilled life of their husband and father.

## Figures and Tables

**Figure 1:**
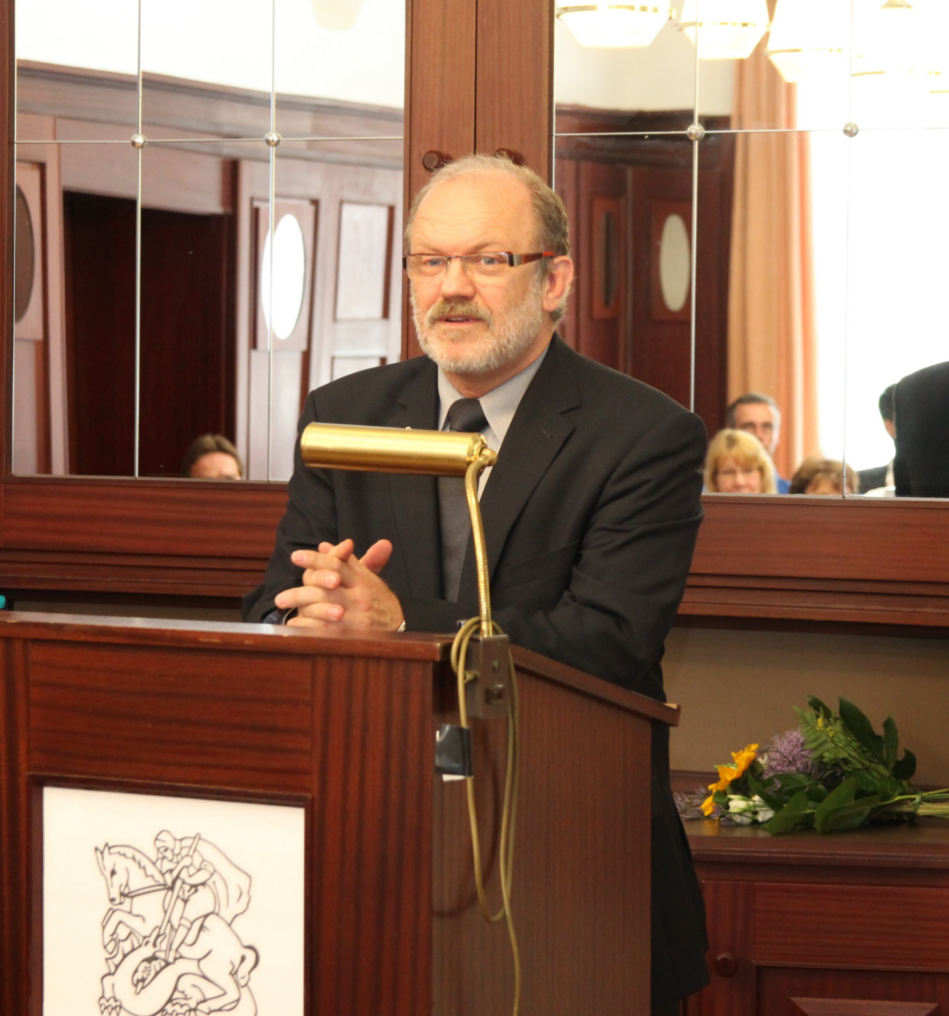
Prof. Dr. Ralf Gahr © Klinikum St. Georg gGmbH, Delitzscher Straße 141, 04129 Leipzig

